# Versatile surface for solid–solid/liquid–solid triboelectric nanogenerator based on fluorocarbon liquid infused surfaces

**DOI:** 10.1080/14686996.2020.1733920

**Published:** 2020-03-05

**Authors:** Jihoon Chung, Handong Cho, Hyungseok Yong, Deokjae Heo, You Seung Rim, Sangmin Lee

**Affiliations:** a School of Mechanical Engineering, Chung-ang University, Seoul, Republic of Korea; b Department of Mechanical Engineering, Mokpo National University, Jeollanam-do, Republic of Korea; c School of Intelligent Mechatronics Engineering, Sejong University, Seoul, Republic of Korea

**Keywords:** Triboelectric nanogenerator, energy harvesting, versatile surface, slips, hierarchical structure, 206 Energy conversion / transport / storage / recovery, 212 Surface and interfaces

## Abstract

The triboelectric nanogenerator (TENG) is a recent mechanical energy harvesting technology that has been attracting significant attention. Its working principle involves the combination of triboelectrification and electrostatic induction. The TENG can harvest electrical energy from both solid–solid and liquid–solid contact TENGs. Due to their physical difference, triboelectric materials in the solid–solid TENG need to have high mechanical properties and the surface of the liquid–solid contact TENG should repel water. Therefore, the surface of the TENG must be versatile for applications in both solid–solid and liquid–solid contact environments. In this work, we develop a solid–solid/liquid–solid convertible TENG that has a slippery liquid-infused porous surface (SLIPS) at the top of the electrode. The SLIPS consists of a HDFS coated hierarchical Al(OH)_3_ structure and fluorocarbon liquid. The convertible TENG developed in this study is capable of harvesting electricity from both solid–solid and liquid–solid contacts due to the high mechanical property of Al(OH)_3_ and the water-based liquid repelling nature of the SLIPS. When the contact occurs in freestanding mode, electrical output was generated through solid–solid/liquid–solid sliding motions. The convertible TENG can harvest electricity from both solid–solid and liquid–solid contacts; thus, it can be a unified solution for TENG surface fabrication.

## Graphical abstract

**Figure:**
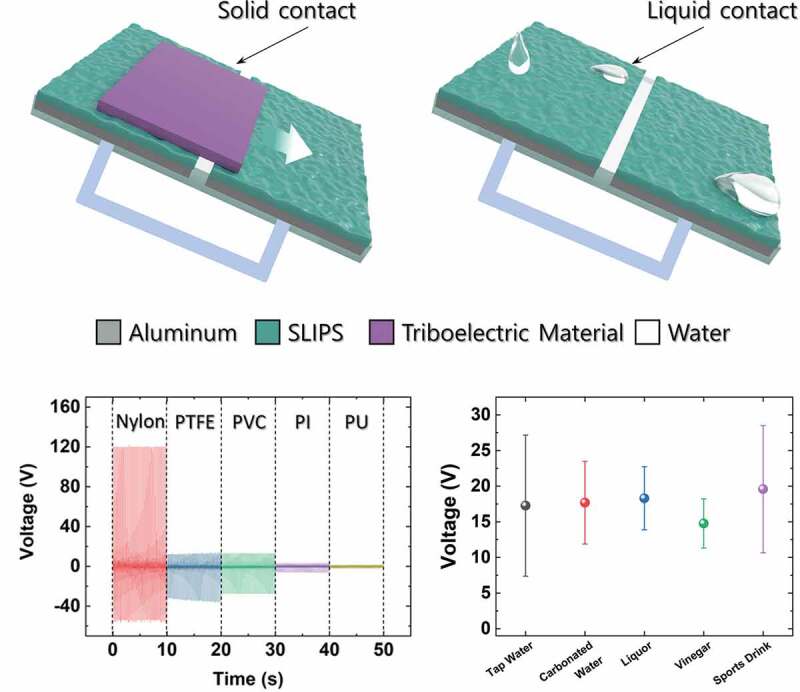

## 1. Introduction

Owing to the rising demand for portable electronics, an increasing number of studies have focused on harvesting electrical energy from ambient sources, including solar [1–3], thermal [4–6], and salinity difference [7–9]. Among these, mechanical energy sources are suitable for harvesting electrical energy since they are less affected by external conditions, such as weather, temperature, and location. Several technologies have been developed for the effective conversion of mechanical energy into electricity, including piezoelectric transducers [10–12], and electromagnetic induction [13–15]. Among these technologies, the triboelectric nanogenerator (TENG), a recently developed mechanical energy harvesting technology, has been attracting significant attention; its working principle is based on the combination of triboelectrification and electrostatic induction [16–19]. In typical TENGs, the electrode is covered with a polymer material to maximize the surface charge, after which it is placed in contact–separation with a counter-charged triboelectric material to generate electricity [20–23]. This counter-charged triboelectric material can be either solid or liquid depending on the working condition [24–27]. Both solid–solid and liquid–solid contact TENGs have distinct characteristics due to their different physical phases. Due to this difference, these two TENGs require different material properties; triboelectric materials in solid–solid TENGs require high mechanical properties for a long lifespan, and the surface of liquid–solid contact TENGs needs to be water repellent for constant liquid separation [28–31]. Previous studies have presented these TENGs as separate devices; therefore, the triboelectric surfaces of TENGs were developed separately as well. However, for a TENG to harvest electricity from ambient mechanical energy sources such as wind and raindrops, it must be able to adapt to both solid–solid and liquid–solid contact environments. Therefore, a unified TENG surface that is capable of effectively harvesting electrical energy from both solid–solid and liquid–solid contacts is required.

In this study, we develop a solid–solid/liquid–solid convertible TENG that has a slippery liquid-infused porous surface (SLIPS) at the top of its electrode. On this device, a low-surface tension fluorocarbon liquid (perfluoropolyether, PFPE, Krytox) was placed over a trichloro(1H,1H,2H,2H-perfluorooctyl) silane (HDFS)-coated Al(OH)_3_ micro-/nanostructure on the aluminum surface. Due to the large number of fluorine atoms on the surface, both the PFPE liquid and HDFS-coated surface can be negatively charged during the contact–separation process, which can lead to the generation of electrical energy from the solid–solid contact. In addition, the surface can effectively repel water-based liquids; thus, it can induce constant contact and separation between the liquid and the TENG surface. The convertible TENG developed in this study could generate electrical energy when in contact with various water-based liquids, such as tap water, carbonated water, liquor, vinegar, and sports drink. When the contact occurred in the freestanding mode, electrical energy was generated from the solid–solid and liquid–solid sliding motions. Thus, this paper presents a unified TENG surface that can effectively harvest electrical energy from both solid–solid and liquid–solid mechanical input.

## 2. Materials and Methods

### 2.1 The fabrication of HDFS coated Al(OH)_3_ surface with PFPE liquid

First, a 1 mm-thick bare aluminum plate was cleaned with deionized water and ethyl alcohol and dried after rinsing. Thereafter, the aluminum plate was dipped into a 0.5 M NaOH (98%, Samchun Chemical, Korea) solution for 1 min and, subsequently, in boiling water for 10 min. To fabricate a hierarchical structure, the aluminum substrate was etched in a 1 M HCl (35–37%, Samchun Chemical, Korea) solution at 80°C for 2 min. The fabricated surface was rinsed with deionized water again and dried at 60°C for 24 h. For the HDFS coating, the aluminum substrate with a hierarchical structure was immersed in a 0.1 v/v% hexane (96%, Samchun Chemical, Korea) solution of HDFS (Gelest, USA) for 10 min at room temperature. Subsequently, the treated sample was thoroughly rinsed with n-hexane and, thereafter, dried at 110°C for 10 min. PFPE liquid was applied on the fabricated aluminum surface and spin-coated at 500 rpm for 1 min. The final fabricated surface was wired and attached to the acrylic substrate for electrical measurement.

### 2.2 Measurements

The electrical measurements including voltage and current measurements were conducted using a mixed domain oscilloscope (MDO 3014, Tektronix Co.) and a low-noise current preamplifier (SR570, Stanford Research Systems Co.). The vertical mechanical input was provided by a vibration tester (ET-126B-4, Labworks Co.) connected to an amplifier (pa-151, Labworks Co.) and function generator (AFG3012C, Tektronix Co.).

### 2.3 Liquid materials

The liquids used in this study are tap water, carbonated water, liquor (Chamisul, 17.8% alcohol, HITEJINRO Co.), vinegar (Apple vinegar, Ottogi Co.), and sports drink (Pocari Sweat, Donga-Otsuka Co.).

## 3. Results and Discussion

**Figure 1.**
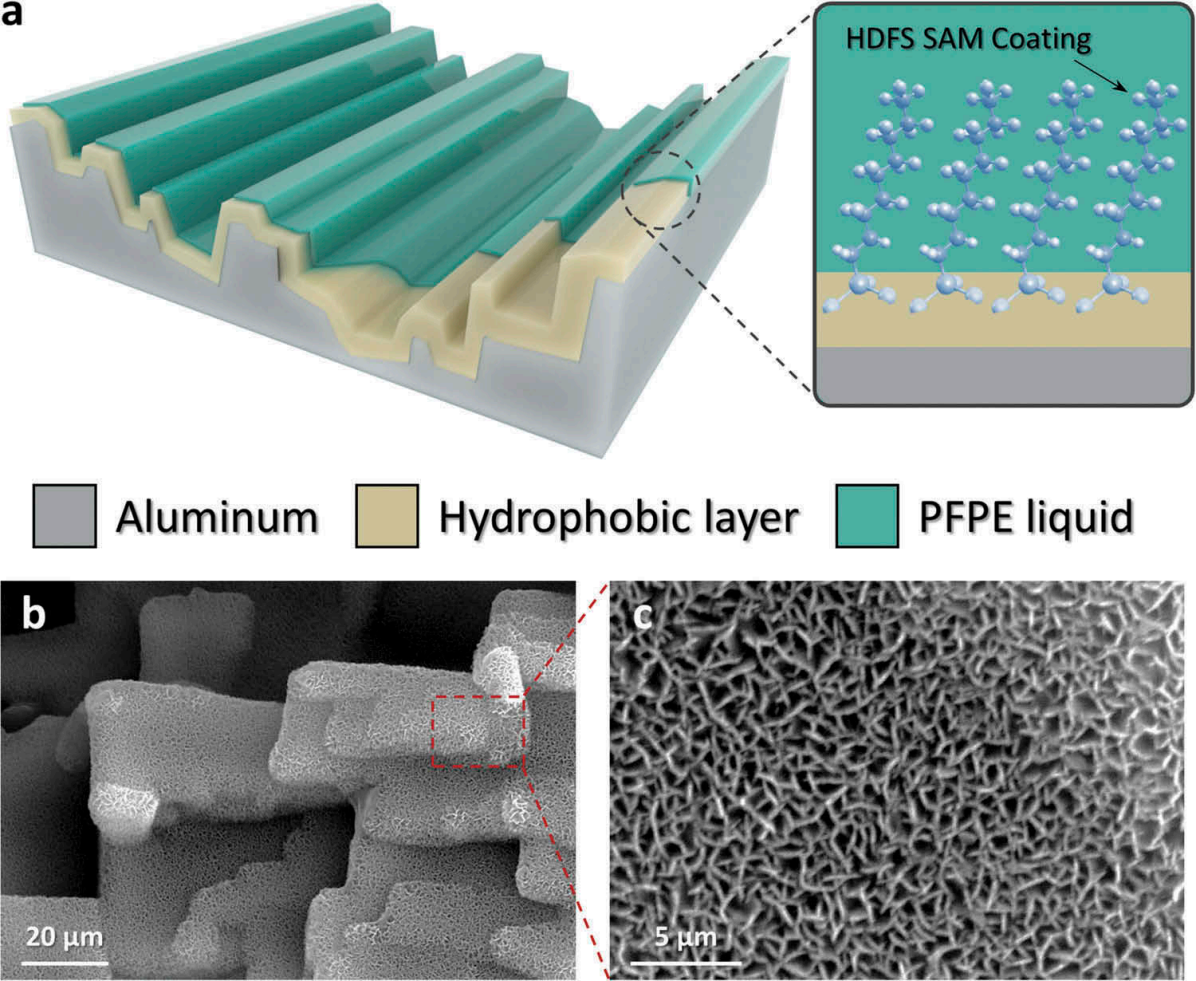
(a) Schematic illustration of the solid–solid/liquid–solid convertible TENG. SAM stands for self-assembled monolayer. Field emission scanning electron microscopy (FE-SEM) image of the (b) microstructures and (c) nanostructures on the aluminum surface

Figure 1 shows the schematic illustration and magnified images of the micro-/nanostructures on the aluminum surface. As shown in Figure 1(a), the hierarchical structure of Al(OH)_3_ is constructed on the aluminum surface. On the outer side of the Al(OH)_3_ layer, a self-assembled monolayer coating of HDFS is fabricated. The PFPE liquid is applied to the hydrophobic hierarchical structure to form a SLIPS. The SLIPS is extremely liquid-repellent, and it can effectively repel water-based liquids [32]. In this study, 1 mL of liquid PFPE is applied to the hierarchical structure and spin-coated for 1 min at 500 rpm to form an evenly distributed thin liquid layer. Figures 1(b,c) and S1 are the magnified images of the hierarchical structure taken by FE-SEM. As shown in the image, both a micro-sized stair-like structure (Figure 1(b)) and a nano-sized wall structure (Figure 1(c)) are formed on the aluminum surface.

In TENGs, selecting a material with a high surface charge is important. A high surface charge will facilitate the flow of electrons, which would, in turn, generate a relatively high electrical output. Generally, materials with a high electron affinity have a corresponding high surface charge. This accounts for the high usage frequency of fluoropolymers, such as polytetrafluoroethylene (PTFE), in TENGs. For comparison with the material used in this device, the PFPE liquid can be expressed as F-(CF(CF_3_)-CF2-O)_n_-CF_2_CF_3_, where n lies within the range of 10–60, and HDFS can be expressed as CF_3_(CF_2_)_5_CH_2_CH_2_SiCl_3_. Both the PFPE liquid and HDFS contain a large number of fluorine atoms that has a high electron affinity. Therefore, the PFPE liquid-applied HDFS surface, which has a high negative surface charge, can be suitable for the solid–solid contact. In addition, the hierarchical structure on top of aluminum is that of Al(OH)_3_, which has more mechanical properties than PTFE [33,34]. The TENG generates electrical output through mechanical contact and friction; consequently, a long lifespan can be expected with high mechanical properties.

**Figure 2.**
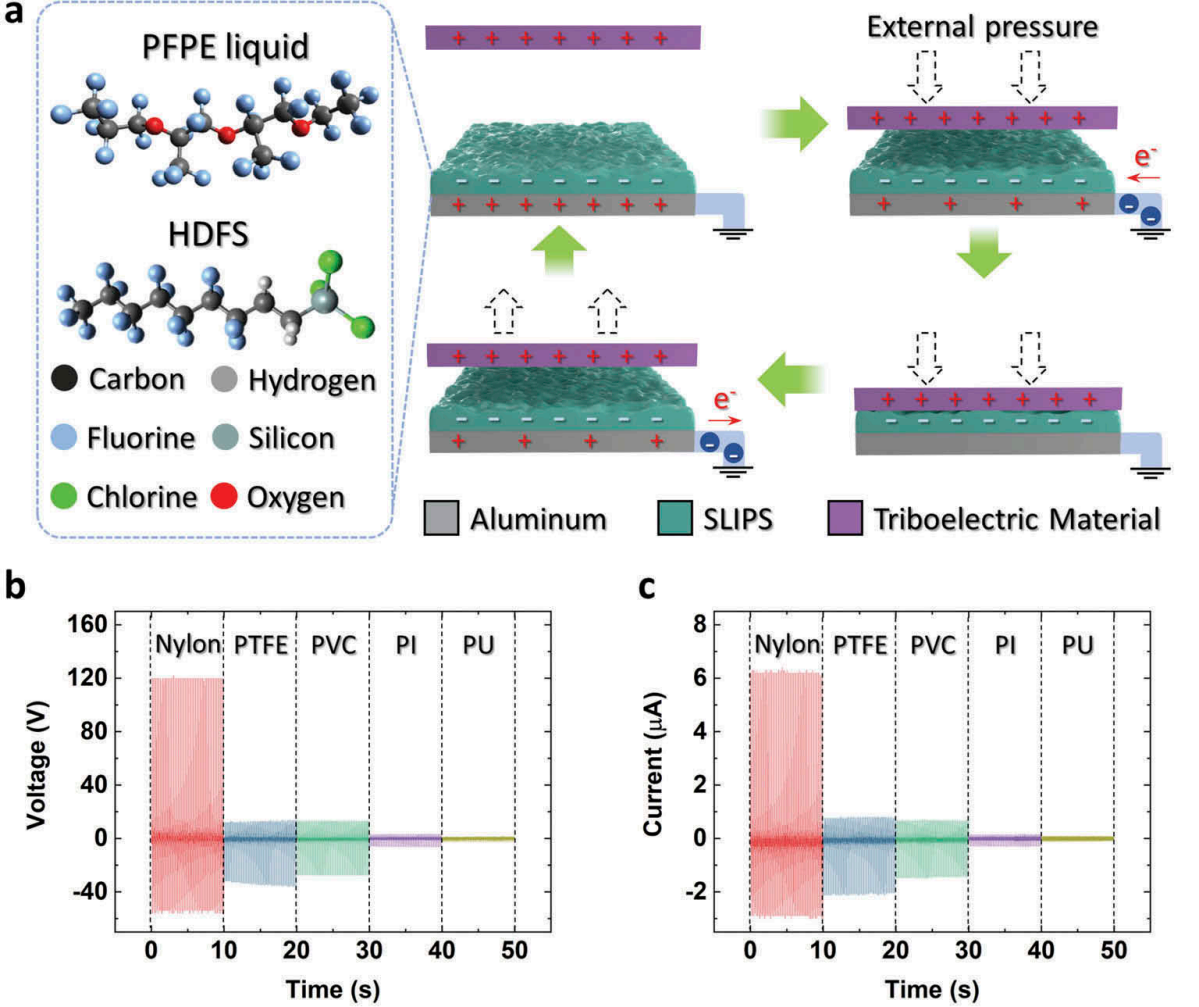
(a) Schematic illustration and working mechanism of the solid–solid contact TENG. (b) *V_OC_* and (c) *I_CC_* outputs of the convertible TENG depending on various materials. PVC, PI and PU stand for polyvinyl chloride, polyimide, and polyurethane, respectively

Figure 2(a) is a schematic illustration of the solid–solid contact TENG working principle, which is the same as that of the single electrode TENG [35,36]. As shown in the figure, the triboelectric material at the top is positively charged and the PFPE liquid-applied HDFS surface is negatively charged due to repeated contact and separation processes. The aluminum electrode at the bottom is affected by the electric field of the SLIPS. As external pressure is applied to the triboelectric material, its surface approaches the single electrode TENG surface. The electrical equilibrium of the aluminum electrode is disrupted by the electric field on the surface of the triboelectric material, and electrons flow into the aluminum electrode. When the triboelectric material contacts the SLIPS, the aluminum electrode attains electrical equilibrium once more. Once the external pressure is eliminated, the triboelectric material detaches from the SLIPS and electrons flow back to the electrical ground owing to the electric field of the SLIPS. By repeating this process, the TENG can produce alternating current (AC) by the contact–separation process between two solid materials.

Figure 2(b,c) show the open-circuit voltage (*V_OC_*) and closed-circuit current (*I_CC_*) outputs of the device, respectively. The TENG was supplied with 6 Hz input using a mechanical vibration tester. As shown in the plot, the TENG generated the highest output when nylon came in contact with the SLIPS. The nylon contact produced high positive peaks, while the PTFE and PVC contacts produced high negative peaks. This is because the PTFE and PVC surfaces became negatively charged when they came in contact with the SLIPS, whereas the nylon surface became positively charged. The SLIPS was formed with the PFPE liquid and HDFS, and for it to produce the highest output when in contact with nylon, it should be negatively charged. When the contact material is nylon, the TENG produces a maximum *V_OC_* of 122 V and a maximum *I_CC_* of 6.4 μA.

**Figure 3.**
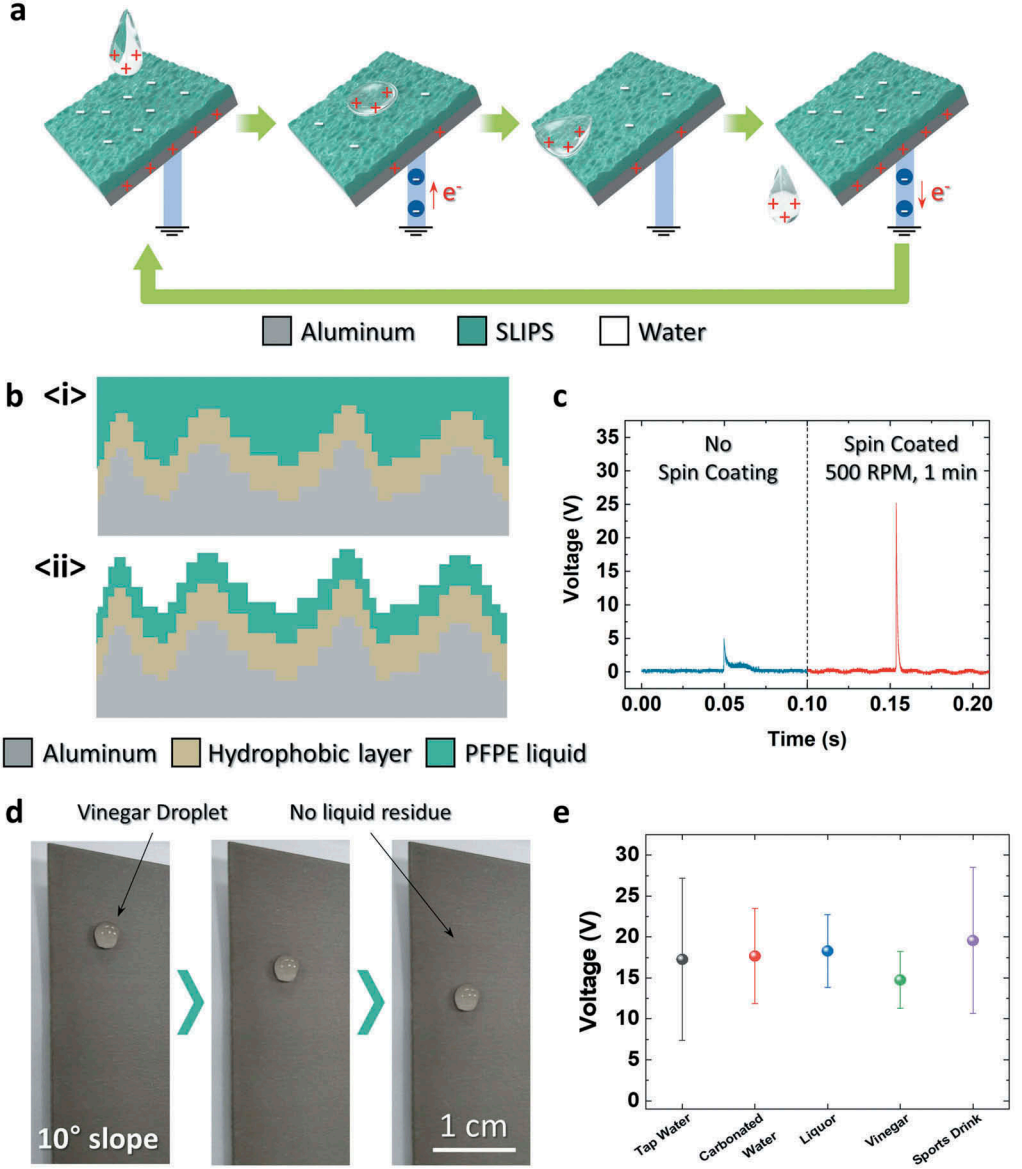
(a) Working mechanism of the liquid–solid contact TENG. (b) Thickness difference of the PFPE liquid depending on the spin-coating process. (c) *V_OC_* output depending on the spin-coating process. (d) Photographs of the vinegar droplet on a 10°-slope SLIPS. (e) Average maximum peak voltage of the liquid–solid contact TENG depending on the liquid

The TENG can generate electrical energy from liquid–solid contact as well because the SLIPS has an excellent liquid repellant property. Figure 3(a) shows the working mechanism of the liquid–solid contact TENG [37,38]. In Figure 3(a), the waterdrop becomes positively charged when it moves through the air and water pipe, and the SLIPS is negatively charged due to the constant contact and separation of the waterdrop. Due to the negatively charged SLIPS, the aluminum electrode will have a positive net charge. When the waterdrop approaches the SLIPS, the positively-charged waterdrop neutralizes the negatively charged SLIPS; therefore, the electrons will flow from the electrical ground to the aluminum electrode. After the waterdrop attaches completely, there will be a minimal surface area difference as the waterdrop moves toward the edge of the TENG. When the waterdrop separates from the TENG, the electrons will flow back to the ground. A repetition of the waterdrop contact and separation processes produces AC.

In a typical TENG, a thin layer of dielectric material is preferred to effectively induce charges on the electrode [39,40]. In this device, the thickness of the dielectric material is equal to the amount of the PFPE liquid remaining on the hierarchical structure. As shown in Figure 3(b–i), the PFPE liquid forms a flat liquid film when initially applied. However, when spin-coated, it forms a thin liquid film along the hierarchical structure on the aluminum electrode (Figure 3(b–ii)). This thickness difference of the PFPE liquid affects the power generation of the device. For comparison, two devices with identical surfaces that have equal amounts of the PFPE liquid were prepared. Subsequently, one sample was spin-coated for 1 min at 500 rpm. Afterward, 2 mL of tap water was dropped on each sample from a height of 20 cm for electrical measurement. As shown in the plot of Figure 3(c), the spin-coated devices produced a peak voltage approximately 5 times higher than that produced by the non-spin-coated device. This indicates that the PFPE liquid is able to properly charge the aluminum electrode when the PFPE liquid film is thin. In addition, the peak-like shape of Al(OH)_3_ hierarchical structure accumulates the electrical charge and enhances the output accordingly.

The SLIPS at the top of the aluminum electrode can repel water-based liquids effectively, including various liquids that are frequently used in everyday life. Figures 3(d) and S2 are photographs of 30 μL-drops of various liquids on a SLIPS, which were taken at 2 s intervals. The surface was tilted 10° for the liquid drop to gravitate toward the edge due to gravitational force. The tested liquids are tap water, carbonated water, liquor (17.8% alcohol), vinegar, and sports drink. As shown in the images, all these liquids slipped to the ground without leaving liquid residues on the surface. Photograph of hierarchical structure without PFPE liquid is shown in Figure S3, after 100 mL of vinegar was poured. As shown in Figure S3, there are many liquid drops pinned on the surface after pouring. These liquid drops left from on the surface would lower the electrical potential difference between liquid and electrode resulting lower output [37]. When 2 mL-liquid drops were dropped from a height of 20 cm, each liquid produced electrical output, as shown in Figure 3(e). The plot represents the maximum peak voltage when each liquid drop was dropped. Although the standard deviations of the voltage peaks are quite large due to the unconstrained nature of the drops, each liquid drop produced 15–20 V on average. This shows the possibility of producing electricity from common used water-based liquids using a SLIPS.

**Figure 4.**
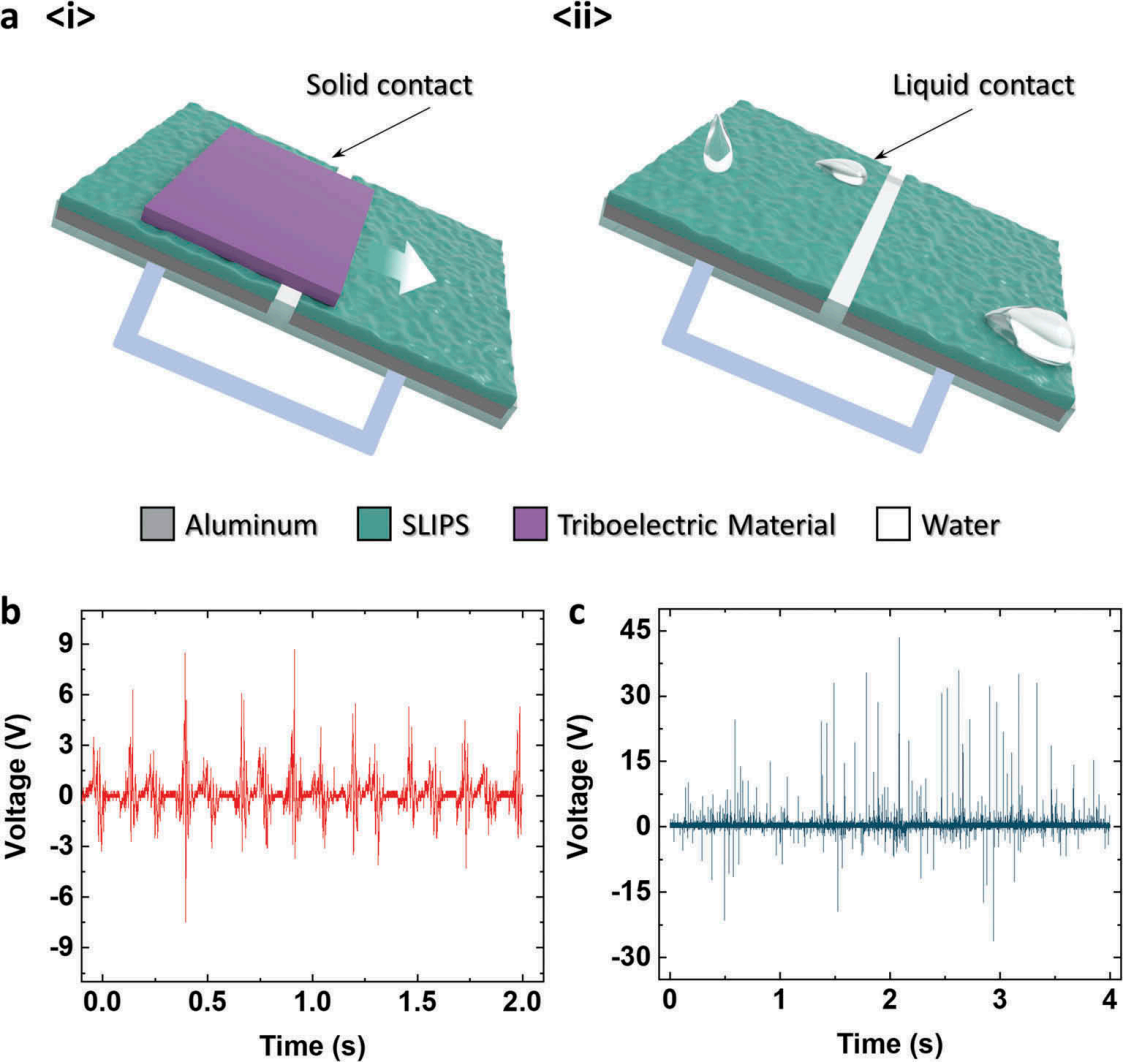
(a) Schematic illustration of the solid–solid/liquid–solid freestanding TENG. *V_OC_* output of the convertible TENG from (b) solid–solid sliding and (c) tap water spraying

A single-electrode-mode TENG discussed in previous paragraphs required an electrical ground for electrons to flow in between. For portable applications, having extra components, such as an electrical ground, can be a critical factor. Therefore, in Figure 4(a), two aluminum electrodes with SLIPSs were attached to an acrylic substrate to generate electrical output in freestanding mode. In the freestanding mode, the TENG can effectively convert sliding mechanical input into electricity. Figure 4(a–i) shows the solid–solid contact freestanding TENG, and Figure 4(a–ii) shows the liquid–solid contact freestanding TENG. For the solid–solid contact TENG, nylon was used as the triboelectric material, and the sliding input was supplied manually (by hand). For the liquid–solid contact TENG, water was sprayed using a commercial shower head. The produced *V_OC_* output is shown in Figure 4(b,c), and the current output is shown in Figure S4. As shown in Figures 4(b) and S4(a), both the *V_OC_* and *I_CC_* show periodic outputs as the solid triboelectric material slides in between two electrodes. In contrast, Figures 4(c) and S4(b) show rather random peak outputs due to the combination of waterdrops falling on to the surface randomly and waterdrops slipping to the ground. The electrical outputs by the solid triboelectric material and liquid drop show their possible application in unified-surface convertible TENGs.

## 4. Conclusions

In summary, we developed a solid–solid/liquid–solid convertible TENG using a PFPE infused surface. Using fluorine abundant materials, the SLIPS could be charged negatively when it came in contact with a counter-charged triboelectric material and utilized in both solid–solid and liquid–solid contact environments. Due to the negatively charged surface, the convertible TENG produced the highest peak *V_OC_* output of 122 V and peak *I_CC_* output of 6.4 μA when the contact material was solid nylon. In addition, the SLIPS on the convertible TENG was capable of repelling water-based liquids. The convertible TENG could produce 15–20 V peak voltages on average using various common used liquids. To demonstrate the applicability of the solid–solid/liquid–solid convertible TENG, a freestanding mode TENG was developed that could harvest electricity from the sliding mechanical motions of both solid and liquid materials. Therefore, the convertible TENG that can harvest electricity from both solid–solid and liquid–solid contacts can be unified solution for TENG surface fabrication.
